# Fur: Find unique genomic regions for diagnostic PCR

**DOI:** 10.1093/bioinformatics/btab059

**Published:** 2021-01-30

**Authors:** Bernhard Haubold, Fabian Klötzl, Lars Hellberg, Daniel Thompson, Markus Cavalar

**Affiliations:** Department of Evolutionary Genetics, Max-Planck-Institute for Evolutionary Biology, Plön, Germany; Department of Evolutionary Genetics, Max-Planck-Institute for Evolutionary Biology, Plön, Germany; Molecular Infection Diagnostics, Euroimmun Medizinische Labordiagnostika, Lübeck, Germany; Molecular Infection Diagnostics, Euroimmun Medizinische Labordiagnostika, Lübeck, Germany; Molecular Infection Diagnostics, Euroimmun Medizinische Labordiagnostika, Lübeck, Germany

## Abstract

**Motivation:**

Unique marker sequences are highly sought after in molecular diagnostics. Nevertheless, there are only few programs available to search for marker sequences, compared to the many programs for similarity search. We therefore wrote the program Fur for Finding Unique genomic Regions.

**Results:**

Fur takes as input a sample of target sequences and a sample of closely related neighbors. It returns the regions present in all targets and absent from all neighbors. The recently published program genmap can also be used for this purpose and we compared it to fur. When analyzing a sample of 33 genomes representing the major phylogroups of *E.coli*, fur was 40 times faster than genmap but used three times more memory. On the other hand, genmap yielded three times more markers, but they were less accurate when tested *in silico* on a sample of 237 *E.coli* genomes. We also designed phylogroup-specific PCR primers based on the markers proposed by genmap and fur, and tested them by analyzing their virtual amplicons in GenBank. Finally, we used fur to design primers specific to a *Lactobacillus* species, and found excellent sensitivity and specificity *in vitro*.

**Availability and implementation:**

Fur sources and documentation are available from https://github.com/evolbioinf/fur. The compiled software is posted as a docker container at https://hub.docker.com/r/haubold/fox.

**Supplementary information:**

Supplementary data are available at *Bioinformatics* online.

## 1 Introduction

Similarity searches as implemented in programs like BLAST and FASTA are among the oldest and most successful computer applications in molecular biology. Given a set of query sequences, we can quickly and reliably find similar regions in sequences collected in a suitable database. In contrast, molecular diagnostics is concerned with *dissimilarity* rather than similarity. Given a set of query or *target* sequences, what are the regions common to them but absent from the genomes of all other organisms? These can then be used to design diagnostic PCR primers.

The answer is usually found by looking for regions shared by all targets and discarding those with similarity to anything else in, say, GenBank. So there are two steps, an intersection step, to identify regions common to all targets, and a subtraction step, to remove non-specific target material.

Both steps can be challenging. Intersection requires a multiple sequence alignment of all targets, which in the case of bacterial genomes is difficult to compute and parse. Similarly, the subsequent subtraction typically results in too many BLAST hits for convenient handling.

**Fig. 1.**
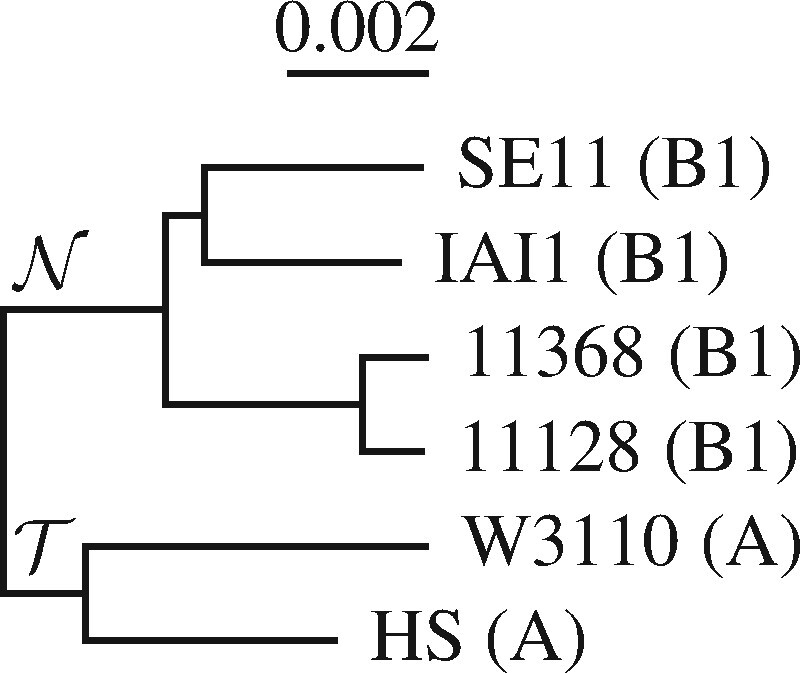
Phylogeny of six *E.coli* strains from phylogroups A and B1. When searching for genetic makers specific for A, T denotes the *targets* and N the *neighbors*

Pockrandt *et al.* (2020) recently published an alternative method for picking markers. Their program, genmap, was originally designed to compute the mappability along a genome. The mappability of a genome position is the inverse of the number of times a fixed-length word, or *k*-mer, starting at it is found elsewhere. Pockrandt *et al.* realized that this could also be used to pick genetic markers given a set of target sequences and their close relatives: Regions with *k*-mers found in all targets but not in the relatives are good marker candidates. The authors demonstrate this by analyzing the full genomes of six *Escherichia coli* strains, two from phylogroup A and four from phylogroup B1 (Fig. 1). Regions with *k*-mers present in strains HS and W3110 but absent from the four other strains would be diagnostic for phylogroup A.

Genmap is designed for quick mappability calculations and requires some programming when repurposed for marker detection. As a more convenient alternative, we wrote a program intended from the start for picking markers. The central idea is that whatever distinguishes a genome from its closest relatives also distinguishes it from every other genome out there. Hence, the subtraction comes first and is done by comparing one of the target sequences to the closest relatives it is to be distinguished from. We call these relatives *neighbors*. Say, we are interested in designing primers specific to phylogroup A in Figure 1, then the members of phylogroup A are the targets, T, the members of phylogroup B1 the neighbors, N.

**Fig. 2.**
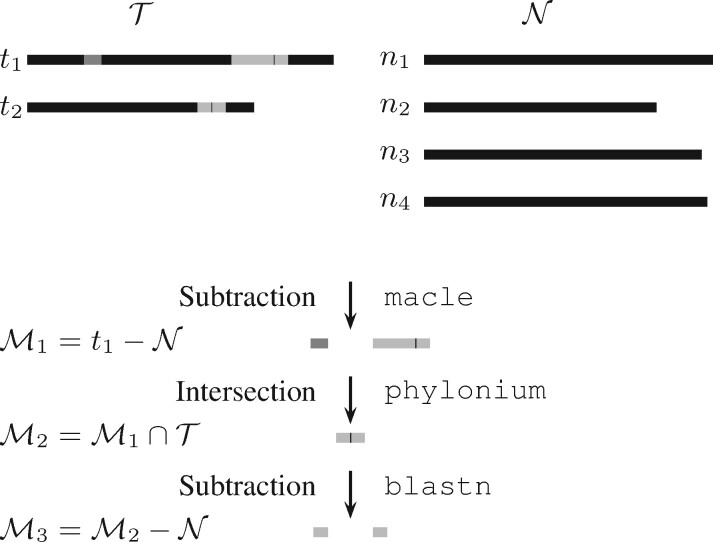
Cartoon of the three steps of the algorithm for marker detection—subtraction, intersection, and again subtraction—applied to two target, T, and four neighbor sequences, N. Here, $M_{1},\;M_{2}$ and $M_{3}$ are the increasingly refined sets of markers for T. Matching shades of gray indicate close homology

In the initial subtraction step, the longest target sequence is compared to the neighbors. This is *t*_1_ in the cartoon of our algorithm in Figure 2. Subtraction of the four neighbors, $n_{1},\ldots ,n_{4}$ leaves the two gray unique regions, one of which still contains a small amount of black non-unique material as the sensitivity of this step is not perfect. Intersecting these two regions with the targets leaves a single marker candidate. In a second and final subtraction step, this marker is again compared to the neighbors and the remaining non-diagnostic material removed.

We call our implementation of this algorithm fur for *Find Unique Regions*. Fur drives three programs, macle, phylonium and BLAST (Fig. 2). Macle was originally written to calculate a measure of sequence complexity based on matches of arbitrary length (Pirogov *et al.*, 2019). Regions of maximal complexity have the same match lengths as random sequences. In other words, maximal complexity indicates absence of close homologs, or presence of uniqueness.

The second program driven by fur, phylonium, was originally written to compute pairwise genetic distances between genomes based on an approximate multiple sequence alignment (Klötzl and Haubold, 2020). This alignment can be used to find regions that occur in every sequence. Macle, phylonium and BLAST are fast programs, which ensures that fur is suitable for investigating samples of whole bacterial genomes on consumer laptops.

In the following, we explain fur in detail. Then we compare it to genmap when applied to simulated and real data. Application to simulated data shows that fur scales gracefully with sequence length and sample size. As real data, we extend the *E.coli* example shown in Figure 1. Richter *et al.* (2018) sequenced the complete genomes of 237 *E.coli* strains sampled from 30 Tanzanian children between 2 and 35 months old. They compared the diversity of this sample to a set of 33 reference *E.coli* and *Shigella* strains, which, in addition to phylogroups A and B1, comprise phylogroups B2, D, E and F. They found that strains turned over quickly and were highly diverse even within individual hosts.

We use this carefully collected data to conduct a training/testing experiment. Fur and genmap are applied to the genomes of the 33 reference strains to identify regions specific to phylogroups. The sensitivity and specificity of these regions is then determined by blasting them against the 237 newly sequenced and classified genomes. This shows that with default settings, genmap is more sensitive and fur more specific. We also calculate a score that combines sensitivity and specificity, which consistently favors fur.

To go from templates to primers, we designed phylogroup-specific primers based on the template sequences identified by genmap and fur, and tested them by looking for *in silico* amplicons in GenBank. All eight primer pairs investigated were highly specific *in silico*.

Finally, we took the project into the lab. Here, our focus was on lactobacilli, which dominate the vaginal microbiota of healthy, pre-menopausal women (Macklaim *et al.*, 2011). Since bacterial vaginosis is associated with a decline in lactobacilli, there is a lot of interest in the possibility of preventing vaginal infections through the use of probiotics (Cribby *et al.*, 2008). The four most common vaginal *Lactobacillus* species are *Liners*, *Lcrispatus*, *Lgasseri* and *Ljensenii*. To demonstrate fur, we constructed primers and probes targeted to *L.crispatus* using genomes representing the other three species as neighbors. When we tested these primer systems on genomic DNA from the four species, they were highly sensitive and specific.

## 2 Materials and methods

### 2.1 Computation

Fur takes as input a database of target and neighbor genomes and returns the regions present in all targets and absent from all neighbors (Fig. 1). These template candidates are found in three steps, subtraction, intersection and again subtraction (Fig. 2). In the first subtraction, a single target is selected and all regions removed that occur anywhere in the neighbors. These are the vast majority of the black regions in Figure 2, leaving the two gray regions with a bit of black. Next, these putatively unique regions are intersected with all targets. In our example, there is only one other target, *t*_2_, which contains no dark gray region, so it is dropped from the candidate set. In addition, the light gray region is truncated. Finally, in the second subtraction, the remaining black part is removed, splitting the light gray segment into the final two marker candidates.

The first subtraction is accomplished by calling an external, single-threaded program, macle, developed by Pirogov *et al.* (2019). Macle computes the match complexity, $C_{m}$, ranging from 0 for regions repeated exactly at least once, to 1 for regions that are effectively random. This complexity can be computed ‘globally’ for an entire sequence, or ‘locally’ for an arbitrary window. When calculated locally, $C_{m}$ amounts to a measure of the genetic distance between the window and its closest homologue anywhere in the sequences analyzed. A distance of 1 indicates the absence of homologues. Under the hood of fur, macle concatenates the forward and reverse strands of all neighbor genomes and of one representative target genome, by default the longest. Then a window of 80 bp is slid along this representative and all overlapping windows with $C_{m}\approx 1$ are merged into non-overlapping regions.

The marker candidates thus obtained need to be checked for presence in all targets, not just the representative. This is done by piling the targets onto the concatenated markers and removing the gapped positions with phylonium running on eight threads (Klötzl and Haubold, 2020). What remains after gap removal are the desired regions present in all targets. These are gap-free, but may contain mismatches.

The second subtraction step is carried out using blastn with eight threads. This is more sensitive but also slower than macle used in the first subtraction. It removes any regions homologous to neighbors that might have been overlooked by macle.

The database queried with fur is constructed using makeFurDb, which is also part of the fur package. It takes as input one directory each of targets and neighbors. It then selects the longest target and merges it with the neighbors into a single macle index, an enhanced suffix array (Ohlebusch, 2013, ch. 4). This index is saved in a new directory, the fur ‘database’. In addition, makeFurDb labels the targets and neighbors before transforming them into a BLAST database, which is also saved in the same directory as the macle index. Once computed, this database can be queried repeatedly using fur with various parameter settings, for example sliding window lengths other than the default 80.

### 2.2 Implementation

Fur and makeFurDb are written in the ‘literate programming’ idiom, where their C-code and documentation are both extracted from a single master file (Knuth, 1992). The package also comprises three auxiliary tools to design and check primers. First, fur2prim converts fur output to primer3 input (Untergasser et al., 2012). Then, prim2fasta extracts the primer sequences from the primer3 results. Finally, checkPrim takes a pair of primers and a BLAST database, and reports *in silico* amplicons. All programs are designed to run under the UNIX command line.

### 2.3 Resource consumption

Simulated data were used to measure the time and memory consumption of genmap and fur. Input consisted of $n_{t}$ targets and $n_{n}$ neighbors, each in its own file. The sequences were simulated as related haplotypes using the coalescent simulator ms (Hudson, 2002) with an expectation of 0.2 mismatches per site, and converted to DNA sequences with ms2dna available from the github repository evolbioinf. The simulation setup was $n_{t}=n_{n}=2$ with lengths varying from 1 Mb to 32 Mb. Both genmap and fur first compute a permanent index, which is then scanned. The fur index is computed by the separate program makeFurDb, while genmap runs in two modes, index and map. In map mode, the word length was 30 with up to 2 mismatches and output was in comma-separated values (CSV) format.

Resource consumption was measured on a laptop equipped with an Intel i7 CPU clocked at 1.8 GHz and 16 GB RAM with Ubuntu 18.04 installed. Run times are ‘user times’.

### 2.4 Data

Three samples of *E.coli*/*Shigella* genomes and one sample of *Lactobacillus* genomes were used in this study: (i) The genomes of the six *E.coli* strains shown in Figure 1 (Supplementary Table S1), five of which are also part of (ii) the 33 *E.coli*/*Shigella* reference strains shown in Figure 6 (Supplementary Table S2), and (iii) the 240 *E.coli* strains sequenced and classified into phylogroups by Richter *et al.* (2018). As reported by these authors, three strains in their collection were not *E.coli*, leaving 237 (Supplementary Table S3). Finally, (iv) the seven *Lactobacillus* genomes underlying the phylogeny in Figure 7 are listed in Supplementary Table S4.

### 2.5 Phylogeny reconstruction

The phylogenies in Figures 1, 6 and 7 were computed from the pairwise number of substitutions per site between their genomes. These were estimated from the full genome sequences using phylonium (Klötzl and Haubold, 2020). The distances were clustered with the neighbor-joining algorithm implemented in clustDist, midpoint rooted with midRoot, and the final tree drawn with new2view. These four programs are also available from the github repository evolbioinf.

### 2.6 Extract markers from genmap output

Given a genmap index, mapping was done with word length 30 and up to 2 errors. The output files in CSV format were scanned with custom scripts for regions where a given word appeared in all targets and in no neighbor.

### 2.7 Primer design

Primers and probes were designed using primer3 (Untergasser *et al.*, 2012) with input generated by fur2prim set to the default parameter values. For each phylogroup, we picked the primer pair with the smallest penalty. It was checked by blasting against a local copy of the non-redundant nucleotide database, nt, downloaded on April 16, 2020. Each accession that generated a virtual amplicon was extracted from the BLAST database. Then the pairwise distances between an accession and the reference sequences were computed with phylonium to determine its phylogroup. This allowed us to measure primer specificity as the number of accessions correctly classified divided by the total number of accessions tested.

### 2.8 Sensitivity and specificity of markers

**Fig. 3.**
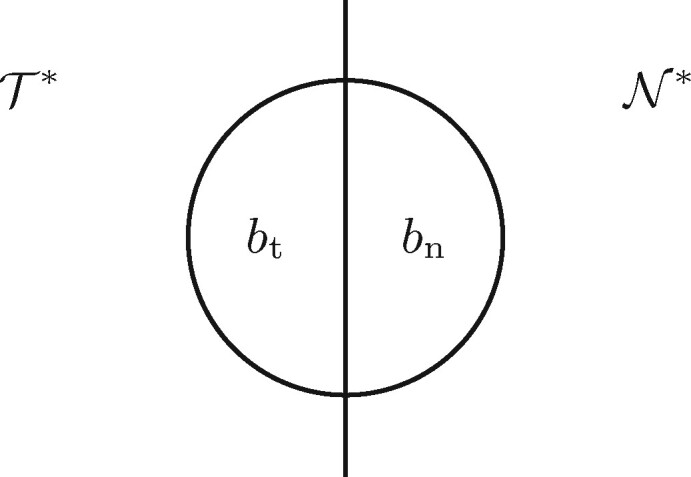
Setup for computing the sensitivity and specificity of a set of unique template sequences uncovered by genmap or fur. $T^{*}$: marker nucleotides in the targets; $N^{*}$: marker nucleotides in the neighbors; $b_{t}$: BLAST hits among targets; $b_{n}$: BLAST hits among neighbors

Sensitivity, specificity and a combined score of a set of marker sequences were computed with the program senSpec—also included with the fur package—following definitions originally developed to assess gene prediction programs (Haubold and Wiehe, 2006, p. 121ff). Figure 3 illustrates the basic setup. Let $T^{*}$ comprise the marker nucleotides that could ideally be found among the targets, and $N^{*}$ the marker nucleotides that could in the worst case be found among the neighbors. For example, if there are two targets, three neighbors, and one 100 bp template, then the size of $T^{*}$ is 200 and the size of $N^{*}$ is 300. Further, let $b_{t}$ be the marker nucleotides actually found by BLAST among the targets, and $b_{n}$ those among the neighbors. Now we define the true positives, $t_{p}=|b_{t}|$ and the false negatives, $f_{n}=|T^{*}-b_{t}|$ to compute the sensitivity,

(1) $$S_{n}=\frac{t_{p}}{t_{p}+f_{n}}.$$

Similarly, let $f_{p}=|b_{n}|$ be the false positives, then the specificity is

(2) $$S_{p}=\frac{t_{p}}{t_{p}+f_{p}}.$$

As good markers are characterized by simultaneously high $S_{n}$ and $S_{p}$, the overall classification quality was measured using the combined score

(3) $$C=\frac{t_{p}t_{n}-f_{p}f_{n}}{\sqrt{\left(t_{p}+f_{p})(t_{n}+f_{n})(t_{n}+f_{p})(t_{p}+f_{n}\right)}},$$

where the true negatives $t_{n}=|N^{*}-b_{n}|$.

### 2.9 *In vitro* analysis

The experimental *Lactobacillus* work was based on the published genomic data from seven strains, three *L.crispatus* targets and neighbors from *L.gasseri*, *L.jensenii* and *L.iners* (Supplementary Table S4). We ran fur on these with BLAST in megablast mode. Given the templates returned, we designed primer pairs and probes, and tested them on a DNA-coupled microarray system similar to that described by Dally *et al.* (2013). As positive control, $∼10^{4}$ copies of genomic DNA from *L.crispatus* served as PCR template. As negative control, $∼10^{6}$ copies of genomic DNA were used as PCR template. The genomic DNA was purchased from the ‘Deutsche Sammlung von Mikroorganismen und Zellkulturen’ (German Collection of Microorganisms and Cellcultures), DSMZ, using the accessions listed in Supplementary Table S5. Note that the training genomes (Supplementary Table S4) are distinct from the testing genomes (Supplementary Table S5).

## 3 Results

### 3.1 Resource consumption

Of the two key computer resources, time and memory, memory imposes the more stringent limit, as a program that does not fit into memory cannot run, regardless of how long we are prepared to wait for its result. So memory consumption is investigated before run time. We do this four times to test fur, makeFurDb and genmap in indexing and mapping modes.

**Fig. 4.**
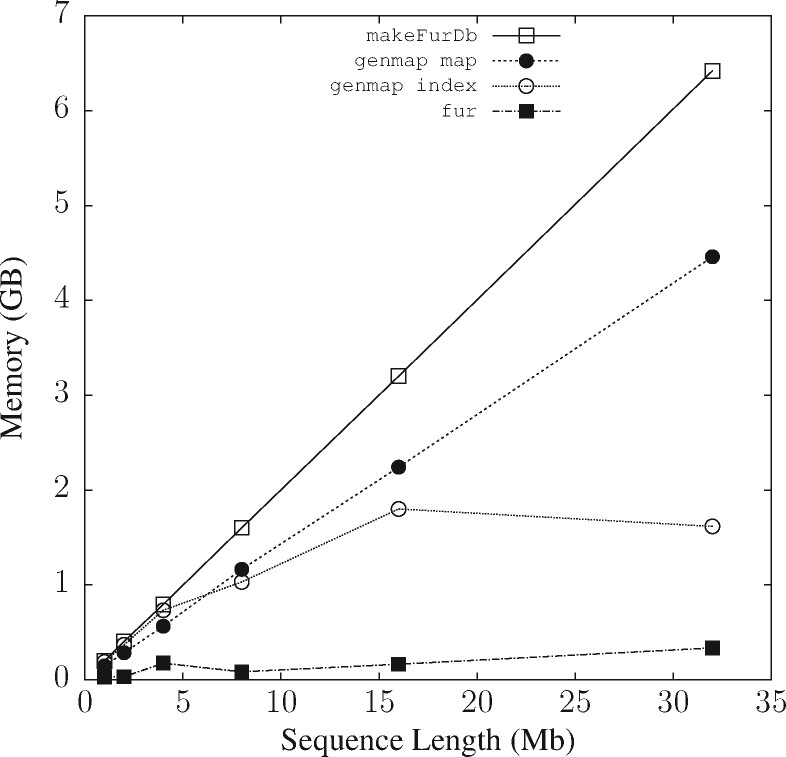
Memory consumption of genmap and makeFurDb/fur as a function of the length of two target and neighbor sequences each

MakeFurDb is the most memory-intensive of the four programs. Its memory requirement is driven by macle, which occupies 70 bytes/bp for indexing. As it indexes three sequences—one target and two neighbors—the memory consumption for sequences 32 Mb long—the longest simulated—is expected to be 3·32·70=6.7 GB, which is close to the 6.4 GB measured for Figure 4. The next most memory-hungry program is the mapping function of genmap, which consumes 35 bytes/bp, or half of what makeFurDb requires. Indexing by genmap is substantially cheaper and appears to be non-linear in the size of the input data occupying 1.6 GB for the total of 32·4=128 Mb input. Similarly, the memory footprint of fur grows very slowly with input size and is by far the smallest of them all with 340 Mb for scanning the macle index of 3·32=96 Mb.

**Fig. 5.**
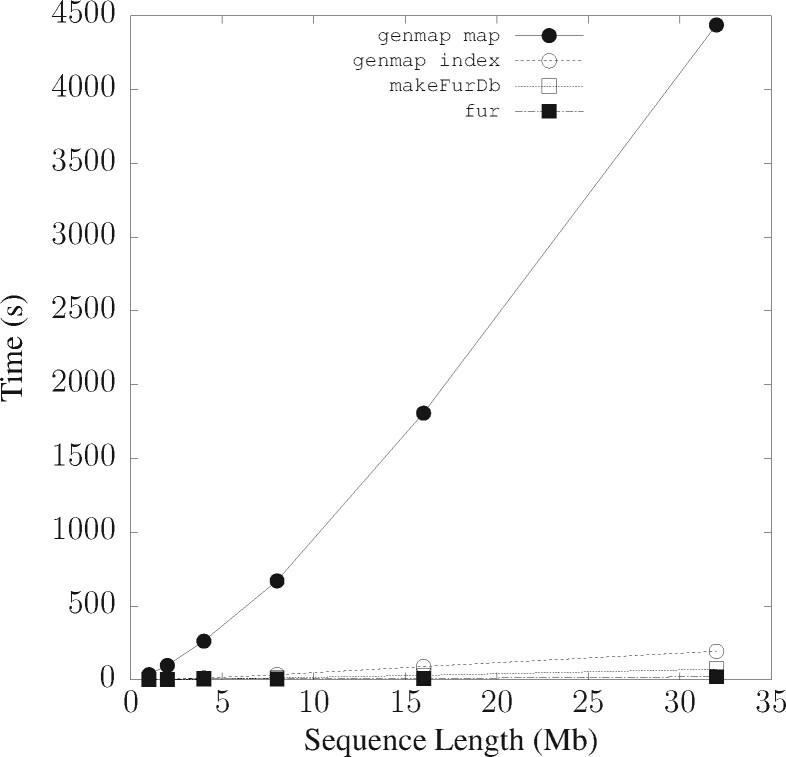
Run time of genmap and makeFurDb/fur as a function of the length of two target and neighbor sequences each

The time consumption of genmap’s mapping function is much larger than that of the other programs and looks non-linear, while indexing takes a mere 1.9 s/Mb (Fig. 5). MakeFurDb is even faster with 0.8 s/Mb and fur the fastest with 0.2 s/Mb.

### 3.2 Phylogroup markers for *E.coli*/*shigella*

**Fig. 6.**
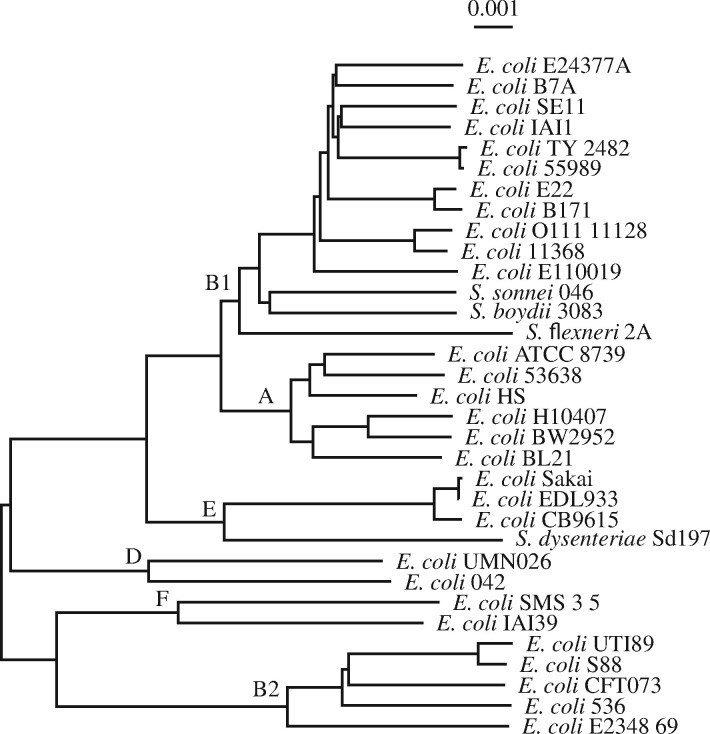
Phylogeny of 33 reference strains of *E.coli*/*Shigella*; the six phylogroups A, B1, B2, D, E and F are marked

Figure 6 shows the phylogeny of the 33 reference *E.coli*/*Shigella* strains used by Richter *et al.* (2018). The genomes are on average 5.1 Mb long totaling 167 Mb. The six phylogroups A, B1, B2, D, E and F are monophyletic. Starting from the top with phylogroup B1, its closest neighbor is phylogroup A, followed by E and then D. Phylogroups F and B2 lie on a separate branch.

We used genmap and fur to identify phylogroup-specific markers, followed by designing phylogroup-specific PCR primers. Since the test set contained no strains belonging to phylogroup F, we restricted our analysis to the five phylogroups A, B1, B2, D and E.

Genmap took roughly 23,000 s, or 6 h 23 min, and 4.1 GB RAM to index and map the reference genomes. The marker yield per phylogroup ranged from 0.7 kb for phylogroup A to 71.4 kb for phylogroup D with an average of 33.6 kb (Table 1). The sensitivity of these regions, tested against the 237 *E.coli* genomes newly sequenced by Richter *et al.* (2018) was generally high, ranging from 0.56 for phylogroup D to a perfect 1 for phylogroup B1 with an average of 0.84 (Table 1). Specificity was less promising, ranging from a very low 0.04 for phylogroup E to 0.6 for phylogroup A with an average of 0.33. The combined score for the genmap markers, *C*, averages 0.34 and ranges from 0.13 in phylogroup E to 0.65 for phylogroup B1. So the 0.9 kb marker sequences for this phylogroup have the best overall power of classification.

**Table 1.** Sensitivity, $S_{n}$, specificity, $S_{p}$ and the combined score, *C*, of genmap marker regions

Phylogroup	Yield (kb)	$S_{n}$	$S_{p}$	*C*
A	0.7	0.99	0.60	0.29
B1	0.9	1.00	0.57	0.65
B2	64.6	0.93	0.25	0.39
D	71.4	0.56	0.21	0.26
E	30.3	0.72	0.04	0.13
Average	33.6	0.84	0.33	0.34

The fur analysis took roughly 540 s, making it 40 times faster than genmap. On the other hand, it occupied 10.6 GB RAM, three times more than genmap. Its yield per phylogroup is ordered as for genmap, except that on average it is three times smaller ranging from no markers for phylogroup A to 22.2 kb for phylogroup D (Table 2). Sensitivity ranges from 0.41 for phylogroup D to 1 for phylogroup B1 and is again sorted in the same order as for genmap, albeit on average smaller, 0.74 compared to 0.84. Specificity ranges from 0.46 for E to 0.97 for B2 and is on average higher than with genmap, 0.74 compared to 0.33. Similarly, the overall score is greater on average, 0.74 compared to 0.34, and ranges from 0.49 for D to 0.99 for B1.

**Table 2.** Sensitivity, $S_{n}$, specificity, $S_{p}$ and the combined score, *C*, of fur marker regions

Phylogroup	Yield (kb)	$S_{n}$	$S_{p}$	*C*
A	0.0	—	—	—
B1	0.1	1.00	0.94	0.99
B2	18.9	0.91	0.97	0.93
D	22.2	0.41	0.65	0.49
E	12.1	0.65	0.46	0.54
Average	13.3	0.74	0.74	0.74

Next, we designed primers for each phylogroup and tested their specificity with respect to GenBank. The best primer pair for the A-markers identified by genmap had a pair-penalty of 0.14 and generated virtual amplicons in a total of 26 GenBank accessions, of which 24 actually belonged to phylogroup A, making these primers reasonably specific (Table 3). Similarly, the primers for phylogroup B1 (488/495), B2 (84/88), D (20/20) and E (168/169) were highly specific, at least *in silico*.

**Table 3.** Phylogroup-specific primers designed from genmap templates

PG	*D*	Sequence	PP	Pos./total
A	f	TCAGCATGGTAGATGCCGTC	0.14	24/26
	r	ATTGCAGGATCAGCACAGCT		
	i	GACGCCCAGCCGCCAGTAAG		
B1	f	CCCGGCCTGTTTATCCATCA	0.22	488/495
	r	ACTGCCCGGTATTCGCTATG		
	i	GCCAGAGTCAAGGGTGTCGGC		
B2	f	AATGGCTTTGGTCAACACGC	0.06	84/86
	r	CAAAAACCGCGGTGTTTTGC		
	i	GCGGTAAATGCTGCCATCGA		
D	f	TTGACGCGTCGTAAACCAGA	0.06	20/20
	r	GAGCCTGATACTCCGTCACG		
	i	ATGCTGCGCAGACGGTGTCC		
E	f	GCGTAACGATAAACGGTGGC	0.06	168/169
	r	CGATGGTCGTCTCCCTTAGC		
	i	GCCGTTGAACAGCCCCAGCA		

*Note*: PG, phylogroup; *D*, direction—forward (f), reverse (r) and internal (i); PP, pair-penalty.

Fur uncovered too little marker material for primer design in phylogroups A and B1 (Table 4). The primers for B2 had a perfect score of 186 out of 186, while the primers for D scored 15/16, and those for E 167/170.

**Table 4.** Phylogroup-specific primers designed from fur templates

PG	*D*	Sequence	PP	Pos./total
A	—	—	—	—
B1	—	—	—	—
B2	f	CGAGTCAGGCGCGTAATACT	0.06	186/186
	r	GCGGATTTGCGCTGATTGAT		
	i	TCGCGATCGCCAGAAAGCCA		
D	f	CACTGATTGCTCGTCATGCG	0.06	15/16
	r	TCGTTGCCCGTTATCAACCA		
	i	GGCCGTTGCGCCCGATTTTG		
E	f	TTGGGTCTGTCATCACCTGC	0.07	167/170
	r	AGCGACGGCGATTACATCAT		
	i	TGCGCTGCACATGCTGACGA		

*Note*: PG, phylogroup; *D*, direction—forward (f), reverse (r) and internal (i); PP, pair-penalty.

### 3.3 *In vitro* analysis of *lactobacillus*

**Fig. 7.**
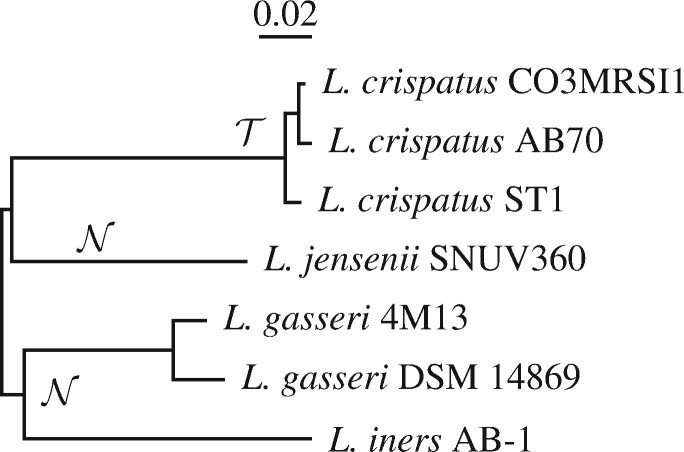
Phylogeny of the seven lactobacilli genomes used for the experimental part of this study. T, targets; N, neighbors

Figure 7 shows the phylogeny of the seven lactobacilli computed from their full genome sequences. There were three *L.crispatus* target genomes, and four neighbor genomes from the three species *L.jensenii*, *L.gasseri* (twice) and *L.iners*. The fur run returned 2908 template fragments totaling 1.1 Mb. From this, we designed primers and probes, of which we tested the four systems listed in Supplementary Table S6. When amplifying DNA from *L.crispatus*—the positive control—the microarray fluorescence was roughly between 62 000 and 64 000 (Table 5). In contrast, when amplifying DNA from *L.gasseri*, *L.iners* or *L.jensenii*—the negative controls—fluorescence ranged from zero to 48, that is over 1200-fold less than in the positive control. In other words, there was virtually no signal from the off-target lactobacilli.

**Table 5.** Microarray fluorescence when amplifying DNA from target and neighbor strains of *Lactobacillus*

		Primer system			
Species	Type	PP100c	PP101	PP103a	PP106
*L.crispatus*	T	62 184	63 703	62 768	63 703
*L.gasseri*	N	16	24	32	8
*L.iners*	N	32	16	32	0
*L.jensenii*	N	16	16	24	48

*Note*: T, target; N, neighbor.

## 4 Discussion

Similarity searches are one of the great success stories of molecular biology and bioinformatics. A short query sequence is submitted to a free program and all similar regions among all published sequences are returned quickly. The frequency with which we carry out such searches can make one forget how remarkable systems like BLAST are. And by taking BLAST for granted, we might expect that its converse—searching for query regions that do not appear in a database—should be similarly straight forward. Such regions are in high demand in molecular diagnostics.

Unfortunately, dissimilarity searches are often much more difficult than similarity searches. For a start, in the age of genomics, we’d like to pinpoint markers across whole genomes, so the query is often a whole genome. Moreover, the target is typically characterized by multiple genomes, not just a single one. So the search might start from all regions conserved among that sample of closely related genomes, a set that is usually not only difficult to determine, but also very large, making subsequent subtraction of non-specific material hard.

These problems are often circumvented by starting from candidate genes. For example, Beghain *et al.* (2018) designed a set of primers for classifying *E.coli* into phylogroups based on *chuA*, among other regions. This gene encodes an outer membrane hemin receptor, which is required for heme transport in certain pathogenic *E.coli* strains (Clermont *et al.*, 2000). While there will always be a place for such a candidate gene approach, a practical, hypothesis-free search for markers across whole genomes is highly desirable.

The authors of genmap noticed that their program—intended for fast mappability computation—could also be used for this purpose (Pockrandt *et al.*, 2020). We had the same aim, using a different approach. Our implementation, fur, is based on two ideas. The first is that subtraction of non-unique material should come *before* the search for conserved regions. This is because there is usually an excess of regions conserved not only in the targets but also in the organisms from which they need to be distinguished (Fig. 2). The first subtraction should be as thorough as possible to facilitate the subsequent steps. This brings us to the second idea, which is that for the subtraction to be maximally effective, it ought to be carried out with respect to the closest relatives that are still distinct. This set of neighbors need not be large. As long as it is closely related to the targets, any target material unique with respect to the neighbors stands a good chance of being unique across the board. This heuristic of course needs to be checked, which is why we went on to quantifying the usefulness of the markers detected with genmap and fur.

As an example analysis, we attempted to design primers specific to *E.coli* phylogroups. Their reliable identification is a long-standing problem in medical diagnostics, as a number of *E.coli* strains are pathogenic in humans, especially in small children. Correspondingly, we took our lead from a study of *E.coli* diversity in 30 Tanzanian children, where Richter *et al.* (2018) had sequenced and classified the genomes of 237 strains. These authors also provided and classified a set of 33 reference strains. We designed markers from the reference set by using members of a phylogroup as targets and all other genomes as their neighbors.

The amount of marker material uncovered with such a design is highly dependent on the genetic distance between targets and neighbors. In the limit of no distance, no markers can be found, as every region in the targets is also present in the neighbors. And, in fact, fur found no markers for A and only 0.1 kb for B1, too little for primer design (Table 2). In contrast, genmap found markers for all five phylogroups, a total of 167.9 Mb (Table 1), roughly three times the 53.3 Mb found by fur.

Good markers are highly sensitive, and in accordance with its greater yield, the average $S_{n}$ for genmap, 0.84, is larger than that of fur, 0.74. The difference in specificity was greater with 0.33 for genmap and 0.74 for fur, leading to an average overall classification quality of 0.33 for genmap compared to 0.74 for fur.

The E templates proposed by genmap had the very low specificity of 0.04. Upon further investigation, we found a large false positive rate, $f_{p}$, inflating the numerator of Equation (2) for calculating $S_{p}$. False positives are BLAST hits among the neighbors (Fig. 3). These depend on the details of the BLAST search. For the computation of $S_{p}$ we used the same BLAST parameters as in the second subtraction step of the fur algorithm (Fig. 2). In particular, by default fur uses the more sensitive blastn mode rather than the megablast mode of standard BLAST. Switching to megablast, which is possible in fur, would drastically alter the specificity of the proposed templates from 0.04 to 0.65 (not shown). Unfortunately, we don’t know which BLAST parameters correspond to PCR-relevant homology, but it might be that with fur we are currently erring on the side of caution.

Having uncovered marker candidates, the work we can do with genmap or fur is done. However, the aim of marker discovery is usually to design diagnostic PCR primers, hence we extended our analysis to also include this step. The eight primer pairs analyzed *in silico* amplified exclusively or almost exclusively accessions belonging to the correct phylogroup, even the primers designed from the E markers uncovered by genmap, which overall had an unusually low specificity (Tables 3 and 4). We did not classify all *E.coli* accessions in GenBank into phylogroups, so we cannot directly quantify the sensitivity of the eight primer pairs investigated. However, it is possible to compare their sensitivity for the three phylogroups B2, D and E with data for both tools. The genmap B2 primers lit up 84 B2 accessions, the fur primers approximately twice as many, 186. The D primers of genmap identified 20 D accessions compared to a quarter fewer, 15, with fur primers. Finally, the E primers based on the two tools identified virtually the same number of E accessions, 168 and 167. Given that fur did not find any usable markers for A and B1, while genmap did, we recommend using fur in a first analysis for its greater speed, specificity and ease with which the templates can be extracted. Should that fail, try genmap for its greater sensitivity.

The logical extension of *in silico* work is to take it into the laboratory. Our *in vitro* analysis was centered on a group of four lactobacilli characteristic of a healthy vaginal microbiome (Fig. 7). We targeted *L.crispatus* and tested the primer systems on three closely related species, *L.gasseri*, *L.iners* and *L.jensenii*, each of them also a common component of vaginal microbiomes. We found a strong signal for the target and virtually none for off-targets (Table 5). Such an outcome is a necessary condition for the primers being useful. Sufficient might be to carry out the negative control on the full diversity of DNA found in the vaginal microbiome, minus *L.crispatus*. We haven’t carried out such a test yet, but plan to analyze vaginal swabs in the future.

In any case, the *in silico* work on *E.coli*/*Shigella* and the *in vitro* work on *Lactobacillus* illustrates that our heuristic—uniqueness with respect to close relatives is equivalent to uniqueness across the board—is useful. This also implies that the results of genmap and fur will depend on the sample of genomes that make up the target and the neighbor sets. Both might need to be revised as new genomes become available.

## Supplementary Material

btab059_Supplementary_Data
