# Supplementary material for: Fur: Find unique genomic regions for diagnostic PCR
Source: Bioinformatics. 2021 Jan 30;37(15):2081–7. doi: 10.1093/bioinformatics/btab059 (PMC8352509; doi:10.1093/bioinformatics/btab059)
Supplement: btab059_Supplementary_Data [file btab059_supplementary_data.pdf]

Supplementary Material for "`Fur`: Find Unique Genomic Regions" by Bernhard Haubold, Fabian Klötzl, Lars Hellberg, Daniel Thompson, and Markus Cavalar

This collection of supplementary material contains five tables describing the three samples of *E. coli*/*Shigella* genomes and two samples of *Lactobacillus* genomes we analyzed. Table S1 contains the phylogroup, strain, accession, and genome length of the six *E. coli* strains whose genomes were used to construct the small phylogeny in Figure 1. Table S2 lists the same information plus the species names of the 33 *E. coli*/*Shigella* strains used by Richter et al. (2018) as reference strains and by us to identify phylogroup-specific markers. Table S3 lists the phylogroup, assembly id, whole genome accession, and genome length of the 237 *E. coli* strains Richter et al. (2018) sequenced and we used to test the quality of the markers uncovered in the reference strains.

As to the *Lactobacillus* data, Table S4 shows the strains whose genomes were used for primer/probe design, and Table S5 the strains whose DNA was used for testing the primer systems experimentally.

Table S1: Strains used to construct the phylogeny in Figure 1

#	Phylogroup	Strain	Accession	Length (Mb)
1	A	HS	CP000802	4.6
2	A	W3110	AP009048	4.6
3	B1	11128	NC_013364	5.4
4	B1	11368	NC_013361	5.7
5	B1	IAI1	CU928160	4.7
6	B1	SE11	AP009240	4.9

Table S2: Strains in reference collection used to construct the phylogeny in Figure 5

#	Phylogroup	Species	Strain	Accession	Length (Mb)
1	A	*E. coli*	53638	NZ_AAKB02000002.1	5.1
2	A	*E. coli*	ATCC_8739	NC_010468.1	4.7
3	A	*E. coli*	BL21	NC_012947.1	4.6
4	A	*E. coli*	BW2952	NC_012759.1	4.6
5	A	*E. coli*	H10407	NC_017633.1	5.2
6	A	*E. coli*	HS	NC_009800.1	4.6
7	B1	*E. coli*	11368	NC_013361.1	5.7
8	B1	*E. coli*	55989	NC_011748.1	5.2
9	B1	*E. coli*	B171	NZ_AAJX02000262.1	5.4
10	B1	*E. coli*	B7A	NZ_CP005998.1	4.9
11	B1	*E. coli*	E110019	CP035751.1	5.2
12	B1	*E. coli*	E22	NZ_AAJV02000126.1	5.5
13	B1	*E. coli*	E24377A	NC_009801.1	5.0
14	B1	*E. coli*	IAI1	NC_011741.1	4.7
15	B1	*E. coli*	O111_11128	NC_013364.1	5.4
16	B1	*E. coli*	SE11	NC_011415.1	4.9
17	B1	*E. coli*	TY_2482	NZ_AFOG01000451.1	5.3
18	B1	*S. boydii*	3083	NC_010658.1	4.6
19	B1	*S. flexneri*	2A	NC_004741.1	4.6
20	B1	*S. sonnei*	046	NC_007384.1	4.8
21	B2	*E. coli*	536	NC_008253.1	4.9
22	B2	*E. coli*	CFT073	AE014075.1	5.2
23	B2	*E. coli*	E2348_69	FM180568.1	5.0
24	B2	*E. coli*	S88	NC_011742.1	5.0
25	B2	*E. coli*	UTI89	NC_007946.1	5.1
26	D	*E. coli*	042	FN554766.1	5.2
27	D	*E. coli*	UMN026	NC_011751.1	5.2
28	E	*E. coli*	CB9615	NC_013941.1	5.4
29	E	*E. coli*	EDL933	NC_002655.2	5.5
30	E	*E. coli*	Sakai	NC_002695.1	5.5
31	E	*S. dysenteriae*	Sd197	NC_007606.1	4.4
32	F	*E. coli*	IAI39	NC_011750.1	5.1
33	F	*E. coli*	SMS_3_5	CP000970.1	5.1

Table S3: The *E. coli* strains sequenced by Richter et al. (2018) used for testing

#	Phylogroup	Assembly	Accession	Length (Mb)
1	A	1-110-08_S1_C1	JHDQ	4.9
2	A	1-110-08_S1_C3	JHDO	5.0
3	A	1-110-08_S3_C1	JHDN	5.5
4	A	1-110-08_S3_C2	JHDM	5.5
5	A	1-110-08_S3_C3	JHDL	5.5
6	A	1-110-08_S4_C2	JHDJ	5.2

Continued on next page

Table S3, continued from previous page

#	Phylogroup	Assembly	Accession	Length (Mb)
7	A	1-110-08_S4_C3	JHDI	5.2
8	A	1-176-05_S1_C1	JHDH	5.1
9	A	1-176-05_S1_C2	JJMA	5.2
10	A	1-176-05_S1_C3	JJLY	5.2
11	A	1-176-05_S3_C1	JHDG	5.3
12	A	1-176-05_S4_C1	JJLQ	5.2
13	A	1-176-05_S4_C2	JMGW	5.1
14	A	1-176-05_S4_C3	JJLI	5.2
15	A	1-182-04_S3_C1	JJLV	5.1
16	A	1-182-04_S3_C2	JJLT	4.6
17	A	1-182-04_S3_C3	JJLS	5.2
18	A	1-250-04_S4_C1	JJLO	5.2
19	A	1-250-04_S4_C2	JJLL	5.2
20	A	1-392-07_S3_C1	JNPY	4.9
21	A	1-392-07_S3_C2	JNPP	4.9
22	A	1-392-07_S3_C3	JJLR	4.9
23	A	2-005-03_S4_C1	JNPS	5.3
24	A	2-011-08_S1_C3	JNNA	5.0
25	A	2-011-08_S3_C1	JNNB	5.2
26	A	2-011-08_S3_C2	JMGU	5.3
27	A	2-011-08_S3_C3	JMGV	5.1
28	A	2-011-08_S4_C1	JNNC	4.8
29	A	2-011-08_S4_C3	JNND	4.8
30	A	2-052-05_S3_C1	JNNF	5.0
31	A	2-052-05_S3_C2	JOSJ	5.0
32	A	2-052-05_S3_C3	JNOZ	4.9
33	A	2-052-05_S4_C3	JNPB	4.9
34	A	2-156-04_S1_C3	JNPE	5.0
35	A	2-156-04_S3_C3	JNPG	5.2
36	A	2-156-04_S4_C1	JNPJ	4.9
37	A	2-156-04_S4_C3	JNQE	4.9
38	A	2-177-06_S3_C2	JNQF	5.4
39	A	2-177-06_S3_C3	JOSO	5.3
40	A	2-210-07_S1_C2	JNPV	5.2
41	A	2-210-07_S1_C3	JNPO	5.2
42	A	2-210-07_S3_C1	JNQH	4.7
43	A	2-210-07_S3_C3	JNQA	5.4
44	A	2-210-07_S4_C2	JNQI	4.9
45	A	2-210-07_S4_C3	JNQJ	4.9
46	A	2-222-05_S3_C1	JOSM	5.5
47	A	2-222-05_S3_C2	JOSN	5.4

Continued on next page

Table S3, continued from previous page

#	Phylogroup	Assembly	Accession	Length (Mb)
48	A	2-222-05_S3_C3	JNQN	5.5
49	A	2-222-05_S4_C1	JORR	4.9
50	A	2-222-05_S4_C2	JNQO	5.3
51	A	2-222-05_S4_C3	JOSP	5.2
52	A	2-316-03_S3_C1	JNQP	5.1
53	A	2-316-03_S4_C2	JNQT	5.3
54	A	2-427-07_S4_C1	JNQZ	5.2
55	A	2-460-02_S3_C1	JNRB	5.5
56	A	2-460-02_S3_C2	JNRC	5.5
57	A	2-460-02_S3_C3	JNRD	5.4
58	A	2-460-02_S4_C1	JONA	5.5
59	A	2-460-02_S4_C2	JNRE	5.3
60	A	2-460-02_S4_C3	JNRF	5.3
61	A	2-474-04_S1_C1	JNRG	5.1
62	A	2-474-04_S1_C2	JNRK	4.9
63	A	2-474-04_S3_C1	JNRH	5.4
64	A	2-474-04_S3_C2	JNRI	5.4
65	A	2-474-04_S3_C3	JNRL	5.4
66	A	3-020-07_S1_C1	JNRO	5.1
67	A	3-020-07_S1_C2	JNRP	5.0
68	A	3-020-07_S1_C3	JNRQ	5.1
69	A	3-020-07_S3_C1	JNRR	5.5
70	A	3-020-07_S3_C2	JONB	5.6
71	A	3-073-06_S1_C2	JNRV	5.2
72	A	3-073-06_S3_C1	JNRW	2.7
73	A	3-073-06_S3_C2	JNRX	5.3
74	A	3-073-06_S4_C1	JNMT	5.4
75	A	3-073-06_S4_C3	JNRZ	5.4
76	A	3-105-05_S1_C1	JNLZ	5.2
77	A	3-105-05_S1_C2	JNSA	5.0
78	A	3-105-05_S1_C3	JNSB	5.2
79	A	3-105-05_S3_C1	JNMA	5.1
80	A	3-105-05_S3_C2	JNMB	5.1
81	A	3-105-05_S3_C3	JNMO	5.0
82	A	3-105-05_S4_C1	JNML	4.9
83	A	3-105-05_S4_C3	JNMD	5.1
84	A	3-267-03_S3_C1	JNMF	5.1
85	A	3-373-03_S1_C1	JNMV	5.1
86	A	3-373-03_S1_C2	JNMI	5.1
87	A	3-373-03_S1_C3	JNMU	5.1
88	A	3-373-03_S3_C1	JNMG	4.6

Continued on next page

Table S3, continued from previous page

#	Phylogroup	Assembly	Accession	Length (Mb)
89	A	3-373-03_S3_C2	JNMQ	4.8
90	A	3-373-03_S3_C3	JNMH	4.9
91	A	3-373-03_S4_C1	JNMW	5.3
92	A	3-373-03_S4_C2	JNMS	5.3
93	A	3-373-03_S4_C3	JOQL	5.3
94	A	3-475-03_S1_C1	JNMJ	4.8
95	A	3-475-03_S4_C1	JNMK	4.9
96	A	3-475-03_S4_C2	JNMX	5.1
97	A	4-203-08_S1_C1	JNMY	5.3
98	A	4-203-08_S1_C2	JOQF	5.3
99	A	4-203-08_S1_C3	JOQG	5.3
100	A	4-203-08_S3_C1	JOQJ	5.0
101	A	4-203-08_S3_C2	JOQK	4.8
102	A	4-203-08_S3_C3	JOQH	4.8
103	A	4-203-08_S4_C2	JOQI	5.1
104	A	4-203-08_S4_C3	JNMZ	5.0
105	A	5-172-05_S1_C3	JOQS	4.9
106	A	5-172-05_S3_C1	JOQQ	5.1
107	A	5-172-05_S3_C3	JOQO	5.2
108	A	5-172-05_S4_C1	JOQP	5.0
109	A	5-172-05_S4_C2	JOQN	5.1
110	A	5-172-05_S4_C3	JOQT	5.0
111	A	5-366-08_S4_C1	JOST	5.6
112	A	5-366-08_S4_C2	JOQM	5.2
113	A	6-175-07_S4_C1	JORB	4.8
114	A	6-175-07_S4_C2	JORC	4.7
115	A	6-175-07_S4_C3	JORK	4.8
116	A	6-319-05_S4_C2	JORH	5.1
117	A	6-319-05_S4_C3	JORJ	5.2
118	A	6-537-08_S1_C1	JORI	5.3
119	A	6-537-08_S1_C2	JOSC	5.3
120	A	6-537-08_S1_C3	JOSF	5.3
121	A	6-537-08_S4_C1	JORS	4.8
122	A	6-537-08_S4_C2	JOSG	4.8
123	A	7-233-03_S1_C2	JORN	4.8
124	A	7-233-03_S1_C3	JORU	4.7
125	A	7-233-03_S3_C1	JORO	5.3
126	A	7-233-03_S3_C2	JORY	5.3
127	A	7-233-03_S3_C3	JORV	5.0
128	A	7-233-03_S4_C1	JOSD	4.7
129	A	7-233-03_S4_C2	JORW	4.8

Continued on next page

Table S3, continued from previous page

#	Phylogroup	Assembly	Accession	Length (Mb)
130	A	7-233-03_S4_C3	JOSE	4.6
131	A	8-415-05_S1_C1	JORZ	4.8
132	A	8-415-05_S1_C2	JOSQ	4.9
133	B1	1-110-08_S1_C2	JHDP	5.0
134	B1	1-182-04_S1_C1	JJMD	5.1
135	B1	1-182-04_S1_C2	JMGS	5.0
136	B1	1-182-04_S1_C3	JJLX	5.0
137	B1	1-182-04_S4_C1	JJLP	5.1
138	B1	1-182-04_S4_C2	JJLM	5.2
139	B1	1-182-04_S4_C3	JJLH	5.1
140	B1	1-392-07_S4_C1	JOSK	5.8
141	B1	1-392-07_S4_C2	JJLK	5.1
142	B1	1-392-07_S4_C3	JOSH	5.8
143	B1	2-005-03_S1_C1	JJME	5.3
144	B1	2-005-03_S1_C2	JJMC	5.3
145	B1	2-005-03_S3_C1	JNPQ	5.3
146	B1	2-005-03_S3_C3	JNPR	5.3
147	B1	2-052-05_S1_C1	JMGR	5.3
148	B1	2-052-05_S1_C3	JNNE	5.2
149	B1	2-052-05_S4_C2	JNPA	5.3
150	B1	2-156-04_S3_C1	JNPF	5.0
151	B1	2-156-04_S4_C2	JNQD	5.1
152	B1	2-177-06_S1_C1	JNPH	5.2
153	B1	2-177-06_S1_C2	JNPL	5.3
154	B1	2-177-06_S1_C3	JNPM	5.2
155	B1	2-177-06_S3_C1	JNPI	5.2
156	B1	2-177-06_S4_C2	JNPN	5.1
157	B1	2-177-06_S4_C3	JNPZ	5.6
158	B1	2-210-07_S3_C2	JNPX	5.2
159	B1	2-210-07_S4_C1	JNPW	5.0
160	B1	2-222-05_S1_C1	JNQK	5.0
161	B1	2-222-05_S1_C2	JNQL	4.9
162	B1	2-222-05_S1_C3	JNQM	4.9
163	B1	2-316-03_S3_C2	JNQQ	5.0
164	B1	2-316-03_S3_C3	JNQR	4.8
165	B1	2-427-07_S1_C1	JNQV	4.7
166	B1	2-427-07_S1_C2	JNQW	4.8
167	B1	2-427-07_S1_C3	JNRJ	5.2
168	B1	2-427-07_S3_C1	JNQX	5.3
169	B1	2-427-07_S3_C3	JNQY	4.9
170	B1	2-427-07_S4_C3	JOMY	5.7

Continued on next page

Table S3, continued from previous page

#	Phylogroup	Assembly	Accession	Length (Mb)
171	B1	2-460-02_S1_C1	JOSS	5.6
172	B1	2-474-04_S4_C1	JOSL	5.0
173	B1	2-474-04_S4_C2	JNRM	5.0
174	B1	2-474-04_S4_C3	JNRN	5.0
175	B1	3-020-07_S4_C2	JNRS	5.3
176	B1	3-020-07_S4_C3	JNRT	5.3
177	B1	3-073-06_S4_C2	JNRY	4.8
178	B1	3-267-03_S1_C1	JNMP	5.5
179	B1	3-267-03_S1_C3	JNME	5.3
180	B1	3-267-03_S3_C2	JNMM	4.9
181	B1	3-267-03_S4_C1	JOND	5.6
182	B1	3-267-03_S4_C2	JNMR	5.4
183	B1	3-475-03_S1_C2	JOQC	5.5
184	B1	3-475-03_S3_C1	JOQD	5.3
185	B1	3-475-03_S3_C2	JOQE	5.4
186	B1	5-366-08_S3_C2	JOQW	5.4
187	B1	6-319-05_S1_C1	JORT	5.2
188	B1	6-319-05_S1_C2	JORD	5.2
189	B1	6-319-05_S1_C3	JORE	5.3
190	B1	6-537-08_S3_C1	JORP	5.2
191	B1	6-537-08_S3_C2	JORX	5.2
192	B1	6-537-08_S3_C3	JORQ	5.2
193	B2	2-005-03_S4_C2	JJLN	5.2
194	B2	2-005-03_S4_C3	JJLJ	5.2
195	B2	2-052-05_S4_C1	JNNG	5.3
196	B2	2-177-06_S4_C1	JNQG	5.1
197	B2	2-316-03_S4_C3	JNQU	5.4
198	B2	2-427-07_S4_C2	JNRA	5.6
199	B2	2-460-02_S1_C2	JOSU	5.7
200	B2	2-460-02_S1_C3	JOMZ	5.5
201	B2	3-020-07_S4_C1	JONC	5.7
202	B2	3-073-06_S1_C1	JNRU	4.9
203	B2	3-105-05_S4_C2	JNMC	5.4
204	B2	6-175-07_S1_C1	JOQR	5.0
205	B2	6-175-07_S3_C1	JOQZ	5.2
206	B2	6-175-07_S3_C2	JONF	5.7
207	B2	6-175-07_S3_C3	JORA	5.4
208	B2	6-319-05_S3_C1	JORF	5.4
209	B2	6-319-05_S3_C2	JORG	5.4
210	B2	6-319-05_S3_C3	JORM	5.3
211	B2	8-415-05_S3_C1	JOSB	5.3

Continued on next page

Table S4: Strain designations and accession numbers for the seven *Lactobacillus* strains used for primer desing.

#	Species	Strain	Class	Length (Mb)	Accession
1	*L. crispatus*	ST1	Target	2.0	NC_014106.1
2	*L. crispatus*	AB70	Target	2.4	NZ_CP026503.1
3	*L. crispatus*	CO3MRSI1	Target	2.3	NZ_CP033426.1
4	*L. gasseri*	DSM 14869	Neighbor	1.9	NZ_CP006803.1
5	*L. gasseri*	4M13	Neighbor	2.1	NZ_CP021427.1
6	*L. jensenii*	SNUV360	Neighbor	1.7	NZ_CP018809.1
7	*L. iners*	AB-1	Neighbor	1.3	NZ_ADHG01

Table S3, continued from previous page

#	Phylogroup	Assembly	Accession	Length (Mb)
212	B2	8-415-05_S3_C2	JOSI	5.3
213	B2	8-415-05_S3_C3	JOSA	5.3
214	B2	8-415-05_S4_C1	JOMT	5.3
215	B2	8-415-05_S4_C2	JOMU	5.2
216	B2	8-415-05_S4_C3	JOMV	5.3
217	D	1-110-08_S4_C1	JHDK	5.5
218	D	1-250-04_S1_C1	JNQB	5.2
219	D	1-250-04_S1_C2	JNQC	5.2
220	D	1-250-04_S1_C3	JJLW	5.1
221	D	1-392-07_S1_C1	JNPT	5.4
222	D	1-392-07_S1_C2	JNPU	5.5
223	D	2-005-03_S3_C2	JMGT	5.3
224	D	2-316-03_S1_C1	JOMW	5.4
225	D	2-316-03_S1_C2	JOMX	5.3
226	D	2-316-03_S4_C1	JNQS	5.2
227	D	3-267-03_S1_C2	JNMN	5.1
228	D	5-366-08_S1_C1	JOQU	5.4
229	D	5-366-08_S1_C3	JONE	5.4
230	D	5-366-08_S3_C1	JOQY	5.3
231	D	5-366-08_S3_C3	JOQV	1.8
232	D	6-175-07_S1_C2	JOMS	5.3
233	D	6-175-07_S1_C3	JORL	5.4
234	E	1-250-04_S3_C1	JJLU	5.1
235	E	1-250-04_S3_C2	JOSR	5.3
236	E	2-005-03_S1_C3	JJLZ	5.3
237	E	2-011-08_S1_C2	JJMB	5.3
238	na	1-176-05_S3_C2	JHDF	4.5
239	na	2-011-08_S1_C1	JMGQ	5.3
240	na	2-156-04_S3_C2	JNPK	5.0

References

T. K. S. Richter, T. H. Hazen, D. Lam, C. L. Coles, J. C. Seidman, Y. You, E. K. Silbergeld, C. M. Fraser, and D. A. Rasko. Temporal variability of *Escherichia coli* diversity in the gastrointestinal tracts of Tanzanian children with and without exposure to antibiotics. *mSphere*, 3:e00558–18, 2018.

Table S5: Strain designations and accesion numbers for the four *Lactobacillus* strains whose genomic DNA was used for *in vitro* primer testing.

#	Species	Control	Strain	DSM-Number
1	*L. crispatus*	positive	VPI 3199	20584
2	*L. jensenii*	negative	62G	20557
3	*L. gasseri*	negative	AM63	20243
4	*L. iners*	negative	—	13335

Table S6: Diagnostic primers and probes for *L. crispatus*.

ID	*D*	Sequence
PP100c	f	CCCGCTATCTCGACTACTGATACAC
	r	GTGTCTTTAGCTAGCTTCCAAACAAA
	i	AGCCAAGCAATATCTCC
PP101	f	GTTTCACAGCAAACCGCTGGG
	r	CGGCTGACTAGCAGGACGAAAA
	i	TGAAAGTAGCCAAGCTC
PP103a	f	GCGTTGTTATATCACACCAAGGCC
	r	GATTCACGAGCCTTGGCGG
	i	TTCTGGTGATTGTGCTAA
PP106	f	GTCCTGGAATTGTCCACCGG
	r	TGATTCACGAGCCTTGGCGG
	i	GTTTATTAATGATCGCTAAGA

D: Direction—forward (f), reverse (r), and internal (i)
